# Public Awareness of Bowel Cancer Risk Factors, Symptoms and Screening in Tasmania, Australia: A Cross-Sectional Study

**DOI:** 10.3390/ijerph19031497

**Published:** 2022-01-28

**Authors:** Simone M. Lee, Vincent L. Versace, Kehinde Obamiro

**Affiliations:** 1 Centre for Rural Health, University of Tasmania, Newnham, TAS 7248, Australia; kehinde.obamiro@utas.edu.au; 2 Deakin Rural Health, Deakin University, Warrnambool, VIC 3280, Australia; vincent.versace@deakin.edu.au

**Keywords:** bowel cancer, colorectal cancer, cancer risk factors, cancer screening, cancer symptoms

## Abstract

Tasmania has one of the highest bowel cancer incidence and death rates in the world. Public awareness of risk factors, symptoms, and early detection of bowel cancer is important for minimising the burden of disease. This study measured awareness levels of bowel cancer risk factors, symptoms and screening in Tasmania. An online survey of 3703 participants aged 18 years and older found that alcohol consumption, low physical activity levels, and having diabetes were the least known risk factors for bowel cancer. Over half of all participants were unaware the risk of bowel cancer increased with age, and 53 percent were not confident they would notice a bowel cancer symptom. Over a third of survey respondents did not know that screening commenced at the age of 50. The results indicate that a targeted campaign to increase bowel cancer awareness in Tasmania may help reduce the high rates of morbidity and mortality from the disease.

## 1. Introduction

Bowel cancer (also known as colorectal cancer) is a global health issue. In 2018 there were an estimated 1.85 million new cases diagnosed, and over 880 thousand recorded deaths [1]. Australia is reported to have the eleventh highest age-standardised incidence rate in the world at 36.9 per 100,000 persons, and the fourth highest death rate from this disease [1]. Among the states and territories of Australia, the island state of Tasmania has the highest incidence rate of bowel cancer (148.9 per 100,000 people aged 50–74) and the fourth highest death rate from the disease (32.0 per 100,000 people aged 50–74) [2]. The reasons why Tasmania has some of the worst bowel cancer figures in the world remains unclear.

Bowel cancer is a highly preventable and treatable condition. Almost half of all bowel cancer cases could be prevented through a healthy lifestyle [3], while ninety-nine percent of cases can be successfully treated if the disease is detected at its earliest stages [4]. Although the age-standardised incidence rate is slowly declining, the total number of new bowel cancer cases in Australia continues to climb [5]. Public awareness of risk factors, symptoms, and early detection of bowel cancer are key factors for minimising the burden of this disease [6]. In Australia, lifestyle risk factors such as obesity [7], alcohol consumption [8], poor diet [9], and low physical activity levels [10] are prevalent, yet screening rates remain low [2], and the proportion of people diagnosed at a late stage remains high [11].

In a recent Australian study, awareness of cancer risk factors was found to be a significant predictor of cancer protective behaviour [12], while not recognising cancer symptoms was reported to be a key reason for delayed patient presentation to a doctor [13,14]. Research also suggests that knowledge of bowel cancer screening predicts greater screening intent [15].

A number of Australian studies have examined population levels of cancer awareness (including bowel cancer) [12,16,17,18], with varying results. To date, no studies have been conducted in Tasmania. In order to better understand the high levels of bowel cancer incidence and death rates in this state, this study aims to measure public awareness levels of bowel cancer risk factors, screening and symptoms in Tasmania.

## 2. Materials and Methods

The study was conducted in Tasmania, Australia. Tasmania is an island state with a population of 534,000 people, of which, 51% are females [19]. The median age in Tasmania is 42 years [19], and 71% of the adult population is considered to be overweight or obese [20]. Based upon the Modified Monash Model classifications (an objective measure of geographical access), the most accessible geographical category in Tasmania is MM2-Regional Centres (i.e., there are no Metropolitan Areas) [21]. More than half of Tasmanian residents (50.24%) reside in areas described as deciles 1–3 (most disadvantaged areas) according to the Australian Bureau of Statistics’ Index of Relative Socio-economic Advantage and Disadvantage (IRSAD) [21,22].

The study was an online survey, promoted using a Facebook (Meta, Menlo Park, CA, USA) advertisement, from which participants were able to click on a link that led to a LimeSurvey page. The Facebook advertisement targeted only people who were 18 years or older and currently residing in Tasmania. The data were cleaned to ensure only those meeting the pre-defined target criteria were included in the analysis. At the start of the survey, participants were able to view an information sheet which provided details about the study. After this, participants were able to provide responses to items on (1) the Bowel Cancer Awareness Measure (Bowel CAM) questionnaire (Version 2.1) [23] modified for online use by Australian participants, and (2) the Cancer Information Overload scale [24]. The Bowel CAM survey instrument was developed by University College London and Cancer Research UK. It is based on a generic CAM developed by Cancer Research UK, University College London, Kings College London and Oxford University in 2007–2008. 

The first part of the survey asked participants to provide sociodemographic and lifestyle information. Location data at the suburb level were used to extract Remoteness Areas, Modified Monash Model data, and Index of Relative Socio-economic Advantage and Disadvantage data. The latter were extracted at Statistical Areas Level 2 (SA2) resolution. SA2s are medium-sized general-purpose areas and are intended to represent a community that interacts together socially and economically [25]. IRSAD is one of four Socio-Economic Indexes for Areas (SEIFA) that are publicly available and are suitable when an analysis requires a general measure of advantage and disadvantage [26].

The second part contained items related to bowel cancer awareness and information overload. A chance to win one of three iPad minis was used to encourage participation. Respondents who did not complete all survey questions were excluded from the data analysis. 

The CAM questionnaires comprise both prompted and unprompted items. Two out of the three unprompted items had a corresponding similar prompted item. Due to the broad range of responses provided for the unprompted items, in combination with the large number of respondents, only data from the prompted items were analysed. Prompted items included awareness of bowel cancer symptoms; risk factors; screening; the relationship between bowel cancer and age; and confidence in identifying bowel cancer symptoms. Items were scored according to the recommendations of the questionnaire developers. For items in the symptom category, ‘no’ and ‘don’t know’ responses were grouped together and scored ‘0’, while ‘yes’ responses were scored ‘1’. For items focusing on risk factors which made use of a Likert scale, ‘strongly agree’ and ‘agree’ responses were grouped together and scored ‘1’; other responses (‘not sure’, ‘disagree’ and ‘strongly disagree’) were scored ‘0’. Correct responses to the item asking about the relationship between bowel cancer and age were scored ‘1’ while incorrect responses were scored ‘0’. A total awareness score was calculated by summing scores from the following questions: symptoms; risk factors; awareness of bowel cancer screening program; and one item asking about the relationship between bowel cancer and age. Responses to the item asking how confident participants were in noticing a bowel cancer symptom were grouped from ‘1’ to ‘4’ (not at all confident to very confident). 

The Cancer Information Overload Questionnaire comprises 8 items measured on a four-point Likert scale (strongly agree to strongly disagree). The total information overload score was determined by summing responses from all the items as described by the scale developers. 

The data were analysed using SPSS version 23 (IBM, Armonk, New York, NY, USA). Categorical variables were analysed using chi-square, while continuous variables were reported as means and standard deviations. Regression analyses were conducted using simple and multiple linear regression. Independent variables with a *p* value of 0.2 or less from the simple regression model were included in the multiple regression analysis. This is because p values between 0.1 and 0.25 have been used as cutoffs in the literature, as there is a tendency for traditional levels such as 0.05 to fail in identifying variables known to be important [27]. The Spearman Correlation Coefficient was used to test for correlations between study variables. For all other analyses, *p* < 0.05 was considered to be statistically significant. 

Ethical approval for the study was granted by the Tasmanian Social Science Human Research Ethics Committee (reference number H0018042). Consent was implied by the submission of the survey responses.

## 3. Results

### 3.1. Sample Characteristics

The process of the Facebook advertisement has been earlier reported and was successful in reaching more than 136,000 people [28]. A total of 4975 respondents across the State filled in both questionnaires. Of this number, 3703 were complete responses and useful for further analysis. The age of participants ranged from 18 to 84 years (median 50 years, IQR 40–60 years), and 76% were females. Sixty-one percent of participants reported having an education below a bachelor’s degree and Caucasian heritage was reported by 95% of respondents. Sixty-eight percent of participants were classified as being overweight or obese, based on self-reported height and weight (Table 1).

### 3.2. Levels of Bowel Cancer Awareness

**Figure 1:**
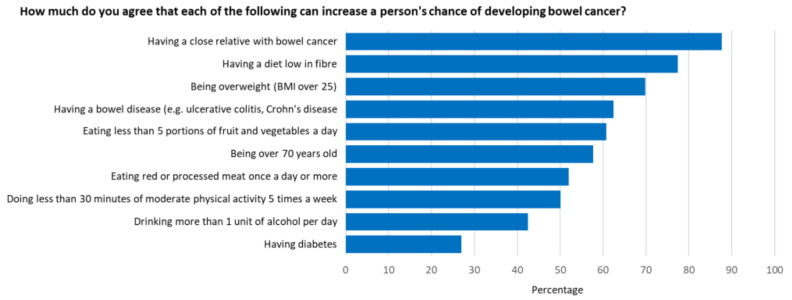
Proportion of participants who agreed or strongly agreed that a given risk factor increased a person’s chance of developing bowel cancer.

**Figure 2:**
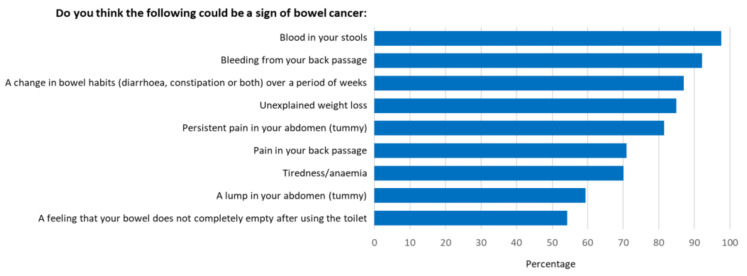
Proportion of participants who recognised prompted symptoms of bowel cancer.

An overall mean awareness score of 14.9 out of 21 (68%) was reported. The survey revealed awareness gaps with regard to several items in both the symptom and risk factor categories. Participants were least aware that low physical activity levels, alcohol consumption and having diabetes were risk factors for bowel cancer (Figure 1), and that tiredness/anaemia, a lump in the abdomen, and a feeling that your bowel does not completely empty after using the toilet were symptoms of bowel cancer (Figure 2). Additionally, 53% did not know that the risk of bowel cancer increases with age; 36% were not aware of the age at which people are first invited for bowel cancer screening in Australia; 53% were not confident that they would be able to notice a bowel cancer symptom. 

### 3.3. Predictors of Bowel Cancer Awareness

Several independent variables were significantly associated with poor bowel cancer awareness in the simple linear regression model (Table 2). This included: education; income; being employed; higher body mass index (BMI); ethnicity; current smoker; and higher perception of information overload. All independent variables with *p* < 0.2 were included in the multiple linear regression model. The results for these factors remained consistent, with the addition of age (Table 3). The multiple linear regression model was significant with F statistics of F(10, 3622) = 41.57, (*p* < 0.001). The Spearman correlation coefficient between variables ranged from 0.002 to 0.422.

## 4. Discussion

Public awareness of bowel cancer in Tasmania was found to be high overall, with an average awareness score of 68%, yet critical gaps were evident around a number of key risk factors, symptoms, and screening. 

Well-recognised risk factors in this study include having a close relative with bowel cancer (88%), a diet low in fibre (78%), and being overweight (70%). Participants were least likely to be aware that alcohol consumption (42%), low physical activity levels (50%) and having diabetes (27%) were risk factors for bowel cancer. 

Underestimating the role of alcohol as a risk factor for cancer is not uncommon. A Western Australian study found alcohol to have low levels of public recognition as a cancer risk factor compared with unestablished/mythic factors such as food additives, stress, and high voltage power lines [16]. In New South Wales, just over half of survey respondents said drinking alcohol contributed to a person’s risk of getting cancer, compared with over 90% for smoking cigarettes [12]. Another Western Australian study with a specific focus on bowel cancer found that only 13% of survey participants who perceived the disease as preventable named drinking alcohol as a risk factor [18]. Similarly, a UK study found that only 19% of respondents said drinking alcohol increased the risk of bowel cancer when unprompted, and 46% when prompted [29]. Yet the role of alcohol in the development of bowel cancer is significant, with 30 grams or more of alcohol per day known to increase the risk [30]. 

In Australia, one in eleven bowel cancer cases (9%) is thought to be attributed to alcohol consumption [31]. This figure is even higher for men than women (12.9% vs. 4.2% respectively), but alcohol is also a risk factor for other cancers including breast, liver, mouth, oesophagus, pharynx and larynx [30]. Combined, 10% of these cancers are thought to be attributed to alcohol consumption [31]. 

To reduce the risk of harm from alcohol-related disease or injury, the National Health and Medical Research Council guidelines state that “healthy men and women should drink no more than 10 standard drinks a week and no more than 4 standard drinks on any one day.” [32]. Yet, 19% of Tasmanians aged 18 years and over are estimated to consume, on average, more than two standard alcoholic drinks per day (or more than 14 standard drinks per week) [33], surpassing current guidelines. Raising public awareness of the cancer risks associated with drinking alcohol may help to reduce high consumption levels, and in turn, the incidence of bowel and other cancers.

While alcohol is an established risk factor for bowel cancer, there is also convincing evidence that physical activity decreases the risk of colon, but not rectal, cancer [30]. Current Australian guidelines recommend adults aged 18–64 years accumulate 150 to 300 minutes of moderate intensity physical activity; or 75 to 150 minutes of vigorous intensity physical activity; or an equivalent combination of both, each week [34]. Benefits for cancer prevention are more likely to be achieved at the higher end of these guidelines, that is, at least 60 minutes of moderate physical activity, 5 times a week [34]. Based on these guidelines, 6.5% of colon cancers, 7.8% of post-menopausal breast cancers, and 6.0% of endometrial cancers can be attributed to insufficient physical activity levels in Australia [35]. The most recent data estimate that the proportion of Australian adults meeting the physical activity guidelines for cancer prevention is only 4% for men and less than 1.0% for women [35]. Given the low awareness levels of the benefits of physical activity for cancer prevention seen in this and other studies [18,29,36,37,38,39], strategies for promoting this message should be encouraged to help reduce the incidence of this disease.

In addition to alcohol consumption and physical inactivity, type 2 diabetes is also considered to be a risk factor for bowel cancer [37,40], with studies demonstrating up to a 30% risk increase [40]. Awareness of this association was very low in our study, with similar results seen in other countries [29,39,41]. While a healthy lifestyle can prevent type 2 diabetes, once established, the condition is difficult to reverse. Messaging should therefore focus on the importance of avoiding type 2 diabetes to reduce the risk of bowel cancer, but also the importance of bowel cancer screening for patients who already have type 2 diabetes.

More than ninety percent of participants recognised that having blood in their stools and bleeding from the back passage were possible signs of bowel cancer. However, tiredness/anaemia (70%), a lump in the abdomen (59%) and a feeling that their bowel does not completely empty after using the toilet (54%), were less likely to be seen as symptoms. These results closely mimic those found by the developers of the Bowel Cancer Awareness Measure used in this study (28). Furthermore, over half of all participants reported they were not confident they would be able to notice a bowel cancer symptom. 

A recent study in Malaysia used the Bowel Cancer Awareness Measure to evaluate the impact of a mass/social media campaign that aimed to raise awareness of bowel cancer symptoms. Two-thirds of those interviewed recalled seeing or hearing the campaign materials on either television (42.9%), indoor/outdoor print (40%) or radio (18.4%) [42]. They found a significant improvement in the recognition of all bowel cancer symptoms at follow-up, compared with pre-campaign awareness levels. Confidence in recognising bowel cancer symptoms also increased significantly at follow-up [42]. These findings support the implementation of a bowel cancer awareness campaign in Tasmania, although robust follow-up of whether greater symptom recognition also resulted in more timely visits to the doctor would also be warranted.

In Australia, the National Bowel Cancer Screening Program (NBCSP) invites people from the age of 50 years to complete a faecal occult blood test biennially. Over a third of survey respondents were unaware that screening commenced at this age. Given that half of these respondents were aged 50 years or less, the importance of raising awareness about bowel cancer screening in this age group cannot be underplayed. In Australia, those aged 50–54 years are consistently less likely to take part in the NBCSP, with current participation rates in this group sitting at 32% compared with the national average of 42% [2]. There is also evidence that those living in rural areas are more likely to have higher rates of bowel cancer and adenoma relative to national statistics [43]. With a third of Tasmania’s population residing in rural and remote areas [44], raising awareness of bowel cancer screening in this population is even more imperative. Research shows that mass media campaigns significantly increase bowel cancer screening in Australia [45,46], with one study finding the relative increase to be greater among those who had never participated in screening. By raising awareness of bowel cancer screening in these younger cohorts, it may be possible to increase first-time participation in the NBCSP. 

From the multiple linear regression model, we found many factors were associated with bowel cancer awareness. Social determinants of health such as education, employment, and income were all positively associated with awareness scores. In contrast, overweight/obesity, current smoker status, and high information overload scores were all negatively associated with awareness scores. 

Studies have found similar results regarding awareness of cancer risk in people with higher BMI levels. A population-based randomised survey of adults aged 50–75 years found obese women were less aware that obesity increased risk for colorectal cancer compared with normal-BMI women [47]. Cardozo et al. [48] found being obese was associated with a lack of knowledge of the effect of obesity on endometrial cancer. Furthermore, an online cross-sectional survey conducted in the UK found obese individuals were less likely to be aware of overweight/obesity as a risk factor for cancer than respondents who were normal weight [49]. Strategies to increase awareness of bowel cancer risk factors, symptoms and screening should therefore take the information needs of overweight and obese individuals into account.

Low cancer awareness has also been found to be common amongst smokers. Current smokers are shown to have less awareness of cancer risk factors [50,51] and cancer symptoms compared with non-smokers [51,52]. Their perceptions of own risk for cancer are also lower [53,54]. Few studies have examined smoking status and bowel cancer awareness; however, a survey conducted in over 10,000 asymptomatic screening participants in Hong Kong found smokers were more likely to have poorer knowledge of bowel cancer symptoms and risk factors compared with non-smokers [55]. Increasing bowel cancer awareness amongst smokers may therefore be a beneficial strategy for reducing morbidity and mortality from the disease.

Participants who reported a high information overload score also tended to have lower awareness scores. This could be because this category of people tends to have poor information-seeking behaviour [56]. Approaches to tackle poor disease awareness should be designed in such a manner that participants do not feel overloaded with information.

## 5. Limitations

Despite the large number of participants in this study, it is apparent that the sample is not representative of the Tasmanian population. The study cohort has a higher proportion of females (76% vs. 51%); a higher median age, driven by sampling those aged 18 years and older (50 years vs. 42 years); and is more educated (39% vs. 16% with a bachelor’s degree or above). In contrast, there was a lower proportion of smokers (9.1% vs. 14.3%) but a similar proportion of people who were overweight or obese compared to Tasmania as a whole (68% vs. 71%). 

According to Eysenbach and Wyatt [57], surveys conducted via the internet are prone to selection bias due to the non-representative nature of the online community, as well as the self-selection of participants, which may explain the results seen. In addition, research has shown that males are less likely to engage with online health-related information, which may go some way to explaining the low participation rates observed in our study [58]. 

Another potential limitation of the study is that symptoms such as abdominal pain, changes in bowel movements, and fatigue are not exclusive to bowel cancer but are common to other bowel conditions such as irritable bowel syndrome [59]. It is possible that awareness of some bowel cancer symptoms may be over- or underestimated in this study, based on participants’ previous knowledge and/or experience with other bowel conditions [60]. 

Finally, developers of the Bowel CAM instrument acknowledge that online surveys are a less controlled environment than face-to-face or telephone surveys, giving participants the opportunity to look up the correct answers or consult with others while completing the survey [23]. Given that the findings of this study closely mimic the results of the original validation study for this instrument [23], the authors are confident that the results accurately represent the awareness of the study cohort. 

## 6. Conclusions

Bowel cancer is a highly preventable and treatable disease when detected early, yet the incidence and death rates from this condition remain high. This study is the first large-scale survey in Tasmania to identify gaps in public awareness of bowel cancer risk factors, symptoms and screening. The results indicate that a targeted campaign to increase bowel cancer awareness may be beneficial, focusing on a broader range of risk factors and symptoms, as well as the eligible screening age for the NBCSP. Evaluation of such a campaign should not only measure changes in awareness, but also consider its impact on prevention, screening, and health-seeking behaviour.

## Figures and Tables

**Table 1:** Demographic characteristics of survey participants.

Parameter	Number and Percentage of Participants—*n* (%)
**Gender**	
Male	892 (24.1)
Female	2806 (75.8)
Other	5 (0.1)
**Age**	
18–49 years	1712 (46.2)
50–74 years	1925 (52.0)
75+ years	61 (1.6)
Missing	5 (0.1)
**Highest education completed**	
Year 12 and below	987 (26.7)
Certificate	649 (17.5)
Diploma	631 (17.0)
Bachelors	1073 (29)
Masters	282 (7.6)
PhD	79 (2.1)
Missing	2 (0.1)
**Current employment status**	
Not currently working	1225 (33.1)
Currently employed	2478 (66.9)
**Annual income bracket (AUD)**	
0 to 18,200	646 (17.5)
18,201 to 37,000	859 (23.2)
37,001 to 90,000	1618 (43.7)
90,001 to 180,000	540 (14.6)
>180,000	38 (1.0)
Missing	2 (0.1)
**Ethnicity**	
Caucasian descent	3504 (94.6)
Non-Caucasian descent	199 (5.4)
**Remoteness area**	
MM2 (regional centres)	2419 (65.4)
MM3 (large rural towns)	557 (15.0)
MM4 (medium rural towns)	7 (0.2)
MM5 (small rural towns)	637 (17.2)
MM6 (remote communities)	47 (1.3)
MM7 (very remote communities)	15 (0.4)
Missing	21 (0.5)
**Index of relative socio-economic advantage and disadvantage**	
1–5 (most disadvantaged)	2460 (66.4)
6–10 (most advantaged)	1233 (33.3)
Missing	10 (0.3)
**Body mass index**	
Under weight	46 (1.2)
Healthy weight	1066 (28.7)
Overweight and obese	2523 (68.1)
Missing	68 (1.8)
**Smoking status**	
Current smoker	333 (9.0)
Non-smoker	3365 (90.9)
Missing	5 (0.1)
**Family history of bowel cancer**	
Positive history	1259 (34.0)
No history	2442 (66.0)
Missing	2 (0.1)

**Table 2:** Factors associated with bowel cancer awareness (simple linear regression model).

Parameter	Estimate	*p* Value
Age (years)	0.007 (−0.002–0.170)	0.123
Gender (female)	0.094 (−0.201–0.389)	0.533
Higher educational level	0.548 (0.470–0.626)	<0.001
Higher income bracket	0.572 (0.441–0.703)	<0.001
Modified Monash Model (increasing rurality) ranking)	0.004 (−0.103–0.110)	0.947
Currently employed	0.897 (0.626–1.168)	<0.001
Ethnicity (non-Caucasian)	−0.626 (−1.195–−0.058)	0.031
BMI (overweight and obese)	−0.662 (−0.939–−0.384)	<0.001
SEIFA IRSAD score	−0.014 (−0.064–0.036)	0.586
Current smoker	−1.052 (−1.497–−0.608)	<0.001
Positive family history of cancer (any type)	0.208 (−0.071–0.486)	0.144
Positive family history of bowel cancer	0.201 (−0.068–0.471)	0.143
Higher information overload	−0.246 (−0.280–−0.213)	<0.001

**Table 3:** Factors associated with bowel cancer awareness (multiple linear regression model).

Variable	B (95% Confidence Interval)	*p* Value
Age	0.010 (0.001–0.019)	0.024
Education level	0.406 (0.324–0.488)	<0.001
Income bracket	0.196 (0.049–0.344)	0.009
Currently employed	0.401 (0.110–0.692)	0.007
Ethnicity (non-Caucasian)	−0.546 (−1.091–0.000)	0.050
Current smoker	−0.535 (−0.965–−0.105)	0.015
BMI (overweight and obese)	−0.339 (−0.607–−0.070)	0.014
Positive family history of cancer (any type)	0.059 (−0.233–0.351)	0.692
Positive family history of bowel cancer	0.229 (−0.053–0.512)	0.112
Information overload score (higher score)	−0.214 (−0.247–−0.181)	<0.001

## Data Availability

The data presented in this study are available on request from the corresponding author. The data are not publicly available due to privacy.
